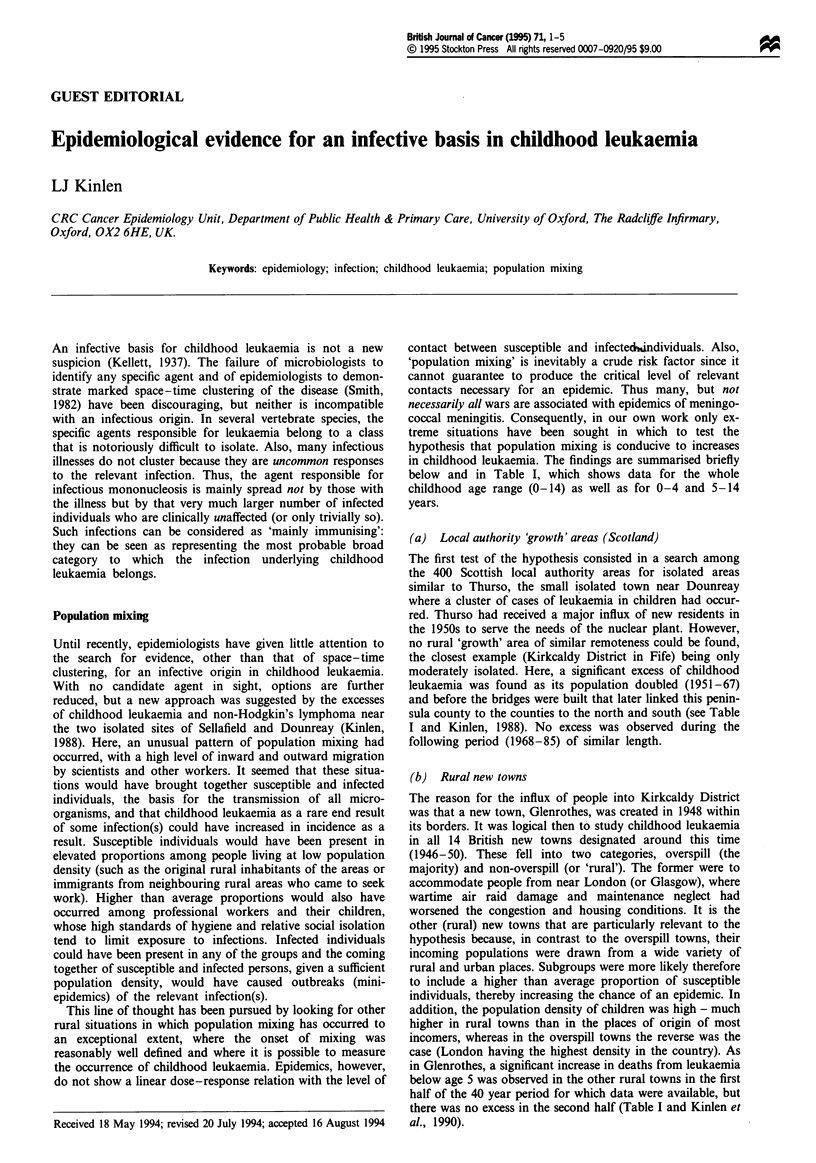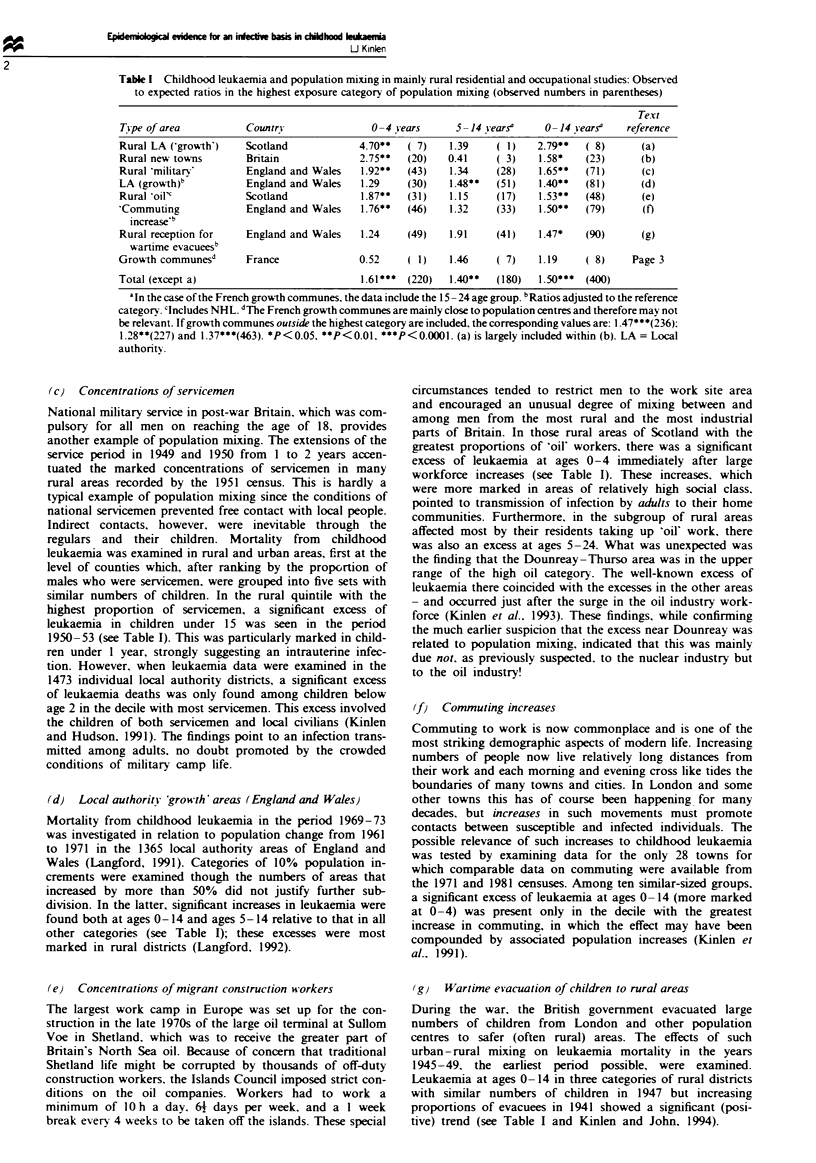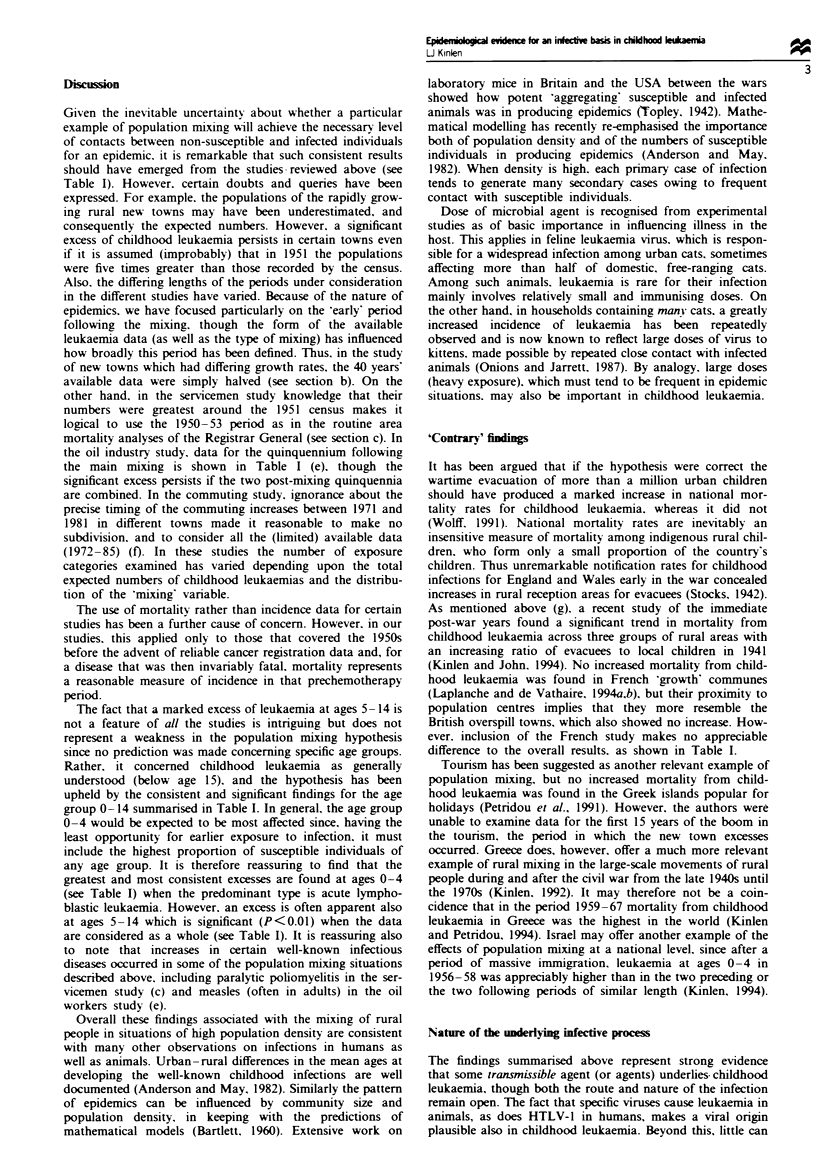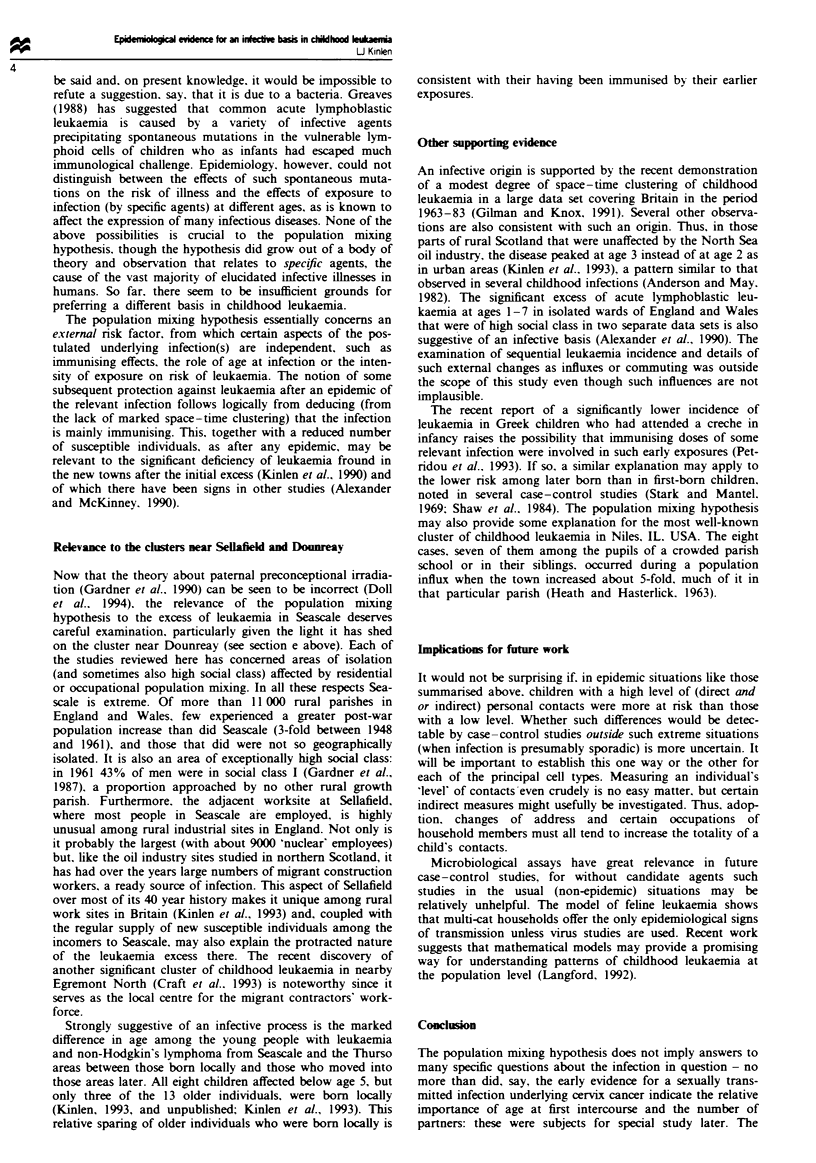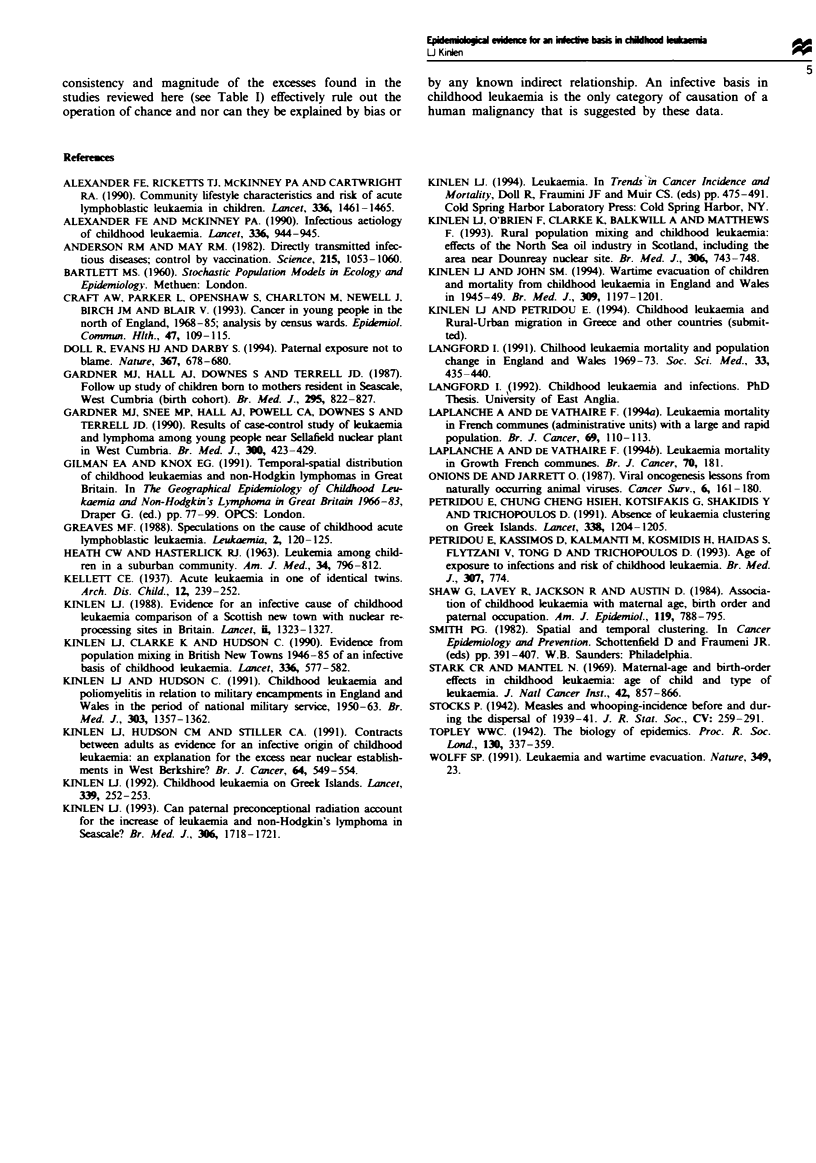# Epidemiological evidence for an infective basis in childhood leukaemia.

**DOI:** 10.1038/bjc.1995.1

**Published:** 1995-01

**Authors:** L. J. Kinlen


British Journal of Cancer (1995) 71, 1-5
© 1995 Stockton Press All rights reserved 0007-0920/95 $9.00

GUEST EDITORIAL

### Epidemiological evidence for an infective basis in childhood leukaemia

LJ Kinlen

*CRC Cancer Epidemiology Unit, Department of Public Health & Primary Care, University of Oxford, The Radcliffe Infirmary, Oxford, OX2 6HE, UK.*

**Keywords**: epidemiology; infection; childhood leukaemia; population mixing

An infective basis for childhood leukaemia is not a new suspicion (Kellett, 1937). The failure of microbiologists to identify any specific agent and of epidemiologists to demonstrate marked space-time clustering of the disease (Smith, 1982) have been discouraging, but neither is incompatible with an infectious origin. In several vertebrate species, the specific agents responsible for leukaemia belong to a class that is notoriously difficult to isolate. Also, many infectious illnesses do not cluster because they are *uncommon* responses to the relevant infection. Thus, the agent responsible for infectious mononucleosis is mainly spread *not* by those with the illness but by that very much larger number of infected individuals who are clinically *un*affected (or only trivially so). Such infections can be considered as 'mainly immunising': they can be seen as representing the most probable broad category to which the infection underlying childhood leukaemia belongs.

**Population mixing**

Until recently, epidemiologists have given little attention to the search for evidence, other than that of space-time clustering, for an infective origin in childhood leukaemia. With no candidate agent in sight, options are further reduced, but a new approach was suggested by the excesses of childhood leukaemia and non-Hodgkin's lymphoma near the two isolated sites of Sellafield and Dounreay (Kinlen, 1988). Here, an unusual pattern of population mixing had occurred, with a high level of inward and outward migration by scientists and other workers. It seemed that these situations would have brought together susceptible and infected individuals, the basis for the transmission of all microorganisms, and that childhood leukaemia as a rare end result of some infection(s) could have increased in incidence as a result. Susceptible individuals would have been present in elevated proportions among people living at low population density (such as the original rural inhabitants of the areas or immigrants from neighbouring rural areas who came to seek work). Higher than average proportions would also have occurred among professional workers and their children, whose high standards of hygiene and relative social isolation tend to limit exposure to infections. Infected individuals could have been present in any of the groups and the coming together of susceptible and infected persons, given a sufficient population density, would have caused outbreaks (mini-epidemics) of the relevant infection(s).

This line of thought has been pursued by looking for other rural situations in which population mixing has occurred to an exceptional extent, where the onset of mixing was reasonably well defined and where it is possible to measure the occurrence of childhood leukaemia. Epidemics, however, do not show a linear dose-response relation with the level of contact between susceptible and infected individuals. Also, 'population mixing' is inevitably a crude risk factor since it cannot guarantee to produce the critical level of relevant contacts necessary for an epidemic. Thus many, but *not necessarily all* wars are associated with epidemics of meningococcal meningitis. Consequently, in our own work only extreme situations have been sought in which to test the hypothesis that population mixing is conducive to increases in childhood leukaemia. The findings are summarised briefly below and in Table I, which shows data for the whole childhood age range (0-14) as well as for 0-4 and 5-14 years.

*(a) Local authority 'growth' areas (Scotland)*

The first test of the hypothesis consisted in a search among the 400 Scottish local authority areas for isolated areas similar to Thurso, the small isolated town near Dounreay where a cluster of cases of leukaemia in children had occurred. Thurso had received a major influx of new residents in the 1950s to serve the needs of the nuclear plant. However, no rural 'growth' area of similar remoteness could be found, the closest example (Kirkcaldy District in Fife) being only moderately isolated. Here, a significant excess of childhood leukaemia was found as its population doubled (1951-67) and before the bridges were built that later linked this peninsula county to the counties to the north and south (see Table I and Kinlen, 1988). No excess was observed during the following period (1968-85) of similar length.

*(b) Rural new towns*

The reason for the influx of people into Kirkcaldy District was that a new town, Glenrothes, was created in 1948 within its borders. It was logical then to study childhood leukaemia in all 14 British new towns designated around this time (1946-50). These fell into two categories, overspill (the majority) and non-overspill (or 'rural'). The former were to accommodate people from near London (or Glasgow), where wartime air raid damage and maintenance neglect had worsened the congestion and housing conditions. It is the other (rural) new towns that are particularly relevant to the hypothesis because, in contrast to the overspill towns, their incoming populations were drawn from a wide variety of rural and urban places. Subgroups were more likely therefore to include a higher than average proportion of susceptible individuals, thereby increasing the chance of an epidemic. In addition, the population density of children was high - much higher in rural towns than in the places of origin of most incomers, whereas in the overspill towns the reverse was the case (London having the highest density in the country). As in Glenrothes, a significant increase in deaths from leukaemia below age 5 was observed in the other rural towns in the first half of the 40 year period for which data were available, but there was no excess in the second half (Table I and Kinlen *et al.*, 1990).

Received 18 May 1994; revised 20 July 1994; accepted 16 August 1994

**Table I** Childhood leukaemia and population mixing in mainly rural residential and occupational studies: Observed to expected ratios in the highest exposure category of population mixing (observed numbers in parentheses)

| *Type of area* | *Country* | *0-4 years* | | *5-14 years*[a] | | *0-14 years*[a] | | *Text reference* |
|---|---|---|---|---|---|---|---|---|
| Rural LA ('growth') | Scotland | 4.70** | ( 7) | 1.39 | ( 1) | 2.79** | ( 8) | (a) |
| Rural new towns | Britain | 2.75** | (20) | 0.41 | ( 3) | 1.58* | (23) | (b) |
| Rural 'military' | England and Wales | 1.92** | (43) | 1.34 | (28) | 1.65** | (71) | (c) |
| LA (growth)[b] | England and Wales | 1.29 | (30) | 1.48** | (51) | 1.40** | (81) | (d) |
| Rural 'oil'[c] | Scotland | 1.87** | (31) | 1.15 | (17) | 1.53** | (48) | (e) |
| 'Commuting increase'[b] | England and Wales | 1.76** | (46) | 1.32 | (33) | 1.50** | (79) | (f) |
| Rural reception for wartime evacuees[b] | England and Wales | 1.24 | (49) | 1.91 | (41) | 1.47* | (90) | (g) |
| Growth communes[d] | France | 0.52 | ( 1) | 1.46 | ( 7) | 1.19 | ( 8) | Page 3 |
| Total (except a) | | 1.61*** | (220) | 1.40** | (180) | 1.50*** | (400) | |

[a]In the case of the French growth communes, the data include the 15-24 age group. [b]Ratios adjusted to the reference category. [c]Includes NHL. [d]The French growth communes are mainly close to population centres and therefore may not be relevant. If growth communes *outside* the highest category are included, the corresponding values are: 1.47***(236); 1.28**(227) and 1.37***(463). $*P<0.05$, $**P<0.01$, $***P<0.0001$. (a) is largely included within (b). LA = Local authority.

*(c) Concentrations of servicemen*

National military service in post-war Britain, which was compulsory for all men on reaching the age of 18, provides another example of population mixing. The extensions of the service period in 1949 and 1950 from 1 to 2 years accentuated the marked concentrations of servicemen in many rural areas recorded by the 1951 census. This is hardly a typical example of population mixing since the conditions of national servicemen prevented free contact with local people. Indirect contacts, however, were inevitable through the regulars and their children. Mortality from childhood leukaemia was examined in rural and urban areas, first at the level of counties which, after ranking by the proportion of males who were servicemen, were grouped into five sets with similar numbers of children. In the rural quintile with the highest proportion of servicemen, a significant excess of leukaemia in children under 15 was seen in the period 1950-53 (see Table I). This was particularly marked in children under 1 year, strongly suggesting an intrauterine infection. However, when leukaemia data were examined in the 1473 individual local authority districts, a significant excess of leukaemia deaths was only found among children below age 2 in the decile with most servicemen. This excess involved the children of both servicemen and local civilians (Kinlen and Hudson, 1991). The findings point to an infection transmitted among adults, no doubt promoted by the crowded conditions of military camp life.

*(d) Local authority 'growth' areas (England and Wales)*

Mortality from childhood leukaemia in the period 1969-73 was investigated in relation to population change from 1961 to 1971 in the 1365 local authority areas of England and Wales (Langford, 1991). Categories of 10% population increments were examined though the numbers of areas that increased by more than 50% did not justify further subdivision. In the latter, significant increases in leukaemia were found both at ages 0-14 and ages 5-14 relative to that in all other categories (see Table I); these excesses were most marked in rural districts (Langford, 1992).

*(e) Concentrations of migrant construction workers*

The largest work camp in Europe was set up for the construction in the late 1970s of the large oil terminal at Sullom Voe in Shetland, which was to receive the greater part of Britain's North Sea oil. Because of concern that traditional Shetland life might be corrupted by thousands of off-duty construction workers, the Islands Council imposed strict conditions on the oil companies. Workers had to work a minimum of 10 h a day, 6½ days per week, and a 1 week break every 4 weeks to be taken off the islands. These special circumstances tended to restrict men to the work site area and encouraged an unusual degree of mixing between and among men from the most rural and the most industrial parts of Britain. In those rural areas of Scotland with the greatest proportions of 'oil' workers, there was a significant excess of leukaemia at ages 0-4 immediately after large workforce increases (see Table I). These increases, which were more marked in areas of relatively high social class, pointed to transmission of infection by *adults* to their home communities. Furthermore, in the subgroup of rural areas affected most by their residents taking up 'oil' work, there was also an excess at ages 5-24. What was unexpected was the finding that the Dounreay-Thurso area was in the upper range of the high oil category. The well-known excess of leukaemia there coincided with the excesses in the other areas - and occurred just after the surge in the oil industry workforce (Kinlen *et al.*, 1993). These findings, while confirming the much earlier suspicion that the excess near Dounreay was related to population mixing, indicated that this was mainly due *not*, as previously suspected, to the nuclear industry but to the oil industry!

*(f) Commuting increases*

Commuting to work is now commonplace and is one of the most striking demographic aspects of modern life. Increasing numbers of people now live relatively long distances from their work and each morning and evening cross like tides the boundaries of many towns and cities. In London and some other towns this has of course been happening for many decades, but *increases* in such movements must promote contacts between susceptible and infected individuals. The possible relevance of such increases to childhood leukaemia was tested by examining data for the only 28 towns for which comparable data on commuting were available from the 1971 and 1981 censuses. Among ten similar-sized groups, a significant excess of leukaemia at ages 0-14 (more marked at 0-4) was present only in the decile with the greatest increase in commuting, in which the effect may have been compounded by associated population increases (Kinlen *et al.*, 1991).

*(g) Wartime evacuation of children to rural areas*

During the war, the British government evacuated large numbers of children from London and other population centres to safer (often rural) areas. The effects of such urban-rural mixing on leukaemia mortality in the years 1945-49, the earliest period possible, were examined. Leukaemia at ages 0-14 in three categories of rural districts with similar numbers of children in 1947 but increasing proportions of evacuees in 1941 showed a significant (positive) trend (see Table I and Kinlen and John, 1994).

### Discussion

Given the inevitable uncertainty about whether a particular example of population mixing will achieve the necessary level of contacts between non-susceptible and infected individuals for an epidemic, it is remarkable that such consistent results should have emerged from the studies reviewed above (see Table I). However, certain doubts and queries have been expressed. For example, the populations of the rapidly growing rural new towns may have been underestimated, and consequently the expected numbers. However, a significant excess of childhood leukaemia persists in certain towns even if it is assumed (improbably) that in 1951 the populations were five times greater than those recorded by the census. Also, the differing lengths of the periods under consideration in the different studies have varied. Because of the nature of epidemics, we have focused particularly on the 'early' period following the mixing, though the form of the available leukaemia data (as well as the type of mixing) has influenced how broadly this period has been defined. Thus, in the study of new towns which had differing growth rates, the 40 years' available data were simply halved (see section b). On the other hand, in the servicemen study knowledge that their numbers were greatest around the 1951 census makes it logical to use the 1950–53 period as in the routine area mortality analyses of the Registrar General (see section c). In the oil industry study, data for the quinquennium following the main mixing is shown in Table I (e), though the significant excess persists if the two post-mixing quinquennia are combined. In the commuting study, ignorance about the precise timing of the commuting increases between 1971 and 1981 in different towns made it reasonable to make no subdivision, and to consider all the (limited) available data (1972–85) (f). In these studies the number of exposure categories examined has varied depending upon the total expected numbers of childhood leukaemias and the distribution of the 'mixing' variable.

The use of mortality rather than incidence data for certain studies has been a further cause of concern. However, in our studies, this applied only to those that covered the 1950s before the advent of reliable cancer registration data and, for a disease that was then invariably fatal, mortality represents a reasonable measure of incidence in that prechemotherapy period.

The fact that a marked excess of leukaemia at ages 5–14 is not a feature of *all* the studies is intriguing but does not represent a weakness in the population mixing hypothesis since no prediction was made concerning specific age groups. Rather, it concerned childhood leukaemia as generally understood (below age 15), and the hypothesis has been upheld by the consistent and significant findings for the age group 0–14 summarised in Table I. In general, the age group 0–4 would be expected to be most affected since, having the least opportunity for earlier exposure to infection, it must include the highest proportion of susceptible individuals of any age group. It is therefore reassuring to find that the greatest and most consistent excesses are found at ages 0–4 (see Table I) when the predominant type is acute lymphoblastic leukaemia. However, an excess is often apparent also at ages 5–14 which is significant ($P < 0.01$) when the data are considered as a whole (see Table I). It is reassuring also to note that increases in certain well-known infectious diseases occurred in some of the population mixing situations described above, including paralytic poliomyelitis in the servicemen study (c) and measles (often in adults) in the oil workers study (e).

Overall these findings associated with the mixing of rural people in situations of high population density are consistent with many other observations on infections in humans as well as animals. Urban–rural differences in the mean ages at developing the well-known childhood infections are well documented (Anderson and May, 1982). Similarly the pattern of epidemics can be influenced by community size and population density, in keeping with the predictions of mathematical models (Bartlett, 1960). Extensive work on laboratory mice in Britain and the USA between the wars showed how potent 'aggregating' susceptible and infected animals was in producing epidemics (Topley, 1942). Mathematical modelling has recently re-emphasised the importance both of population density and of the numbers of susceptible individuals in producing epidemics (Anderson and May, 1982). When density is high, each primary case of infection tends to generate many secondary cases owing to frequent contact with susceptible individuals.

Dose of microbial agent is recognised from experimental studies as of basic importance in influencing illness in the host. This applies in feline leukaemia virus, which is responsible for a widespread infection among urban cats, sometimes affecting more than half of domestic, free-ranging cats. Among such animals, leukaemia is rare for their infection mainly involves relatively small and immunising doses. On the other hand, in households containing *many* cats, a greatly increased incidence of leukaemia has been repeatedly observed and is now known to reflect large doses of virus to kittens, made possible by repeated close contact with infected animals (Onions and Jarrett, 1987). By analogy, large doses (heavy exposure), which must tend to be frequent in epidemic situations, may also be important in childhood leukaemia.

### 'Contrary' findings

It has been argued that if the hypothesis were correct the wartime evacuation of more than a million urban children should have produced a marked increase in national mortality rates for childhood leukaemia, whereas it did not (Wolff, 1991). National mortality rates are inevitably an insensitive measure of mortality among indigenous rural children, who form only a small proportion of the country's children. Thus unremarkable notification rates for childhood infections for England and Wales early in the war concealed increases in rural reception areas for evacuees (Stocks, 1942). As mentioned above (g), a recent study of the immediate post-war years found a significant trend in mortality from childhood leukaemia across three groups of rural areas with an increasing ratio of evacuees to local children in 1941 (Kinlen and John, 1994). No increased mortality from childhood leukaemia was found in French 'growth' communes (Laplanche and de Vathaire, 1994*a*,*b*), but their proximity to population centres implies that they more resemble the British overspill towns, which also showed no increase. However, inclusion of the French study makes no appreciable difference to the overall results, as shown in Table I.

Tourism has been suggested as another relevant example of population mixing, but no increased mortality from childhood leukaemia was found in the Greek islands popular for holidays (Petridou *et al*., 1991). However, the authors were unable to examine data for the first 15 years of the boom in the tourism, the period in which the new town excesses occurred. Greece does, however, offer a much more relevant example of rural mixing in the large-scale movements of rural people during and after the civil war from the late 1940s until the 1970s (Kinlen, 1992). It may therefore not be a coincidence that in the period 1959–67 mortality from childhood leukaemia in Greece was the highest in the world (Kinlen and Petridou, 1994). Israel may offer another example of the effects of population mixing at a national level, since after a period of massive immigration, leukaemia at ages 0–4 in 1956–58 was appreciably higher than in the two preceding or the two following periods of similar length (Kinlen, 1994).

### Nature of the underlying infective process

The findings summarised above represent strong evidence that some *transmissible* agent (or agents) underlies childhood leukaemia, though both the route and nature of the infection remain open. The fact that specific viruses cause leukaemia in animals, as does HTLV-1 in humans, makes a viral origin plausible also in childhood leukaemia. Beyond this, little can be said and, on present knowledge, it would be impossible to refute a suggestion, say, that it is due to a bacteria. Greaves (1988) has suggested that common acute lymphoblastic leukaemia is caused by a variety of infective agents precipitating spontaneous mutations in the vulnerable lymphoid cells of children who as infants had escaped much immunological challenge. Epidemiology, however, could not distinguish between the effects of such spontaneous mutations on the risk of illness and the effects of exposure to infection (by specific agents) at different ages, as is known to affect the expression of many infectious diseases. None of the above possibilities is crucial to the population mixing hypothesis, though the hypothesis did grow out of a body of theory and observation that relates to *specific* agents, the cause of the vast majority of elucidated infective illnesses in humans. So far, there seem to be insufficient grounds for preferring a different basis in childhood leukaemia.

The population mixing hypothesis essentially concerns an *external* risk factor, from which certain aspects of the postulated underlying infection(s) are independent, such as immunising effects, the role of age at infection or the intensity of exposure on risk of leukaemia. The notion of some subsequent protection against leukaemia after an epidemic of the relevant infection follows logically from deducing (from the lack of marked space–time clustering) that the infection is mainly immunising. This, together with a reduced number of susceptible individuals, as after any epidemic, may be relevant to the significant deficiency of leukaemia fround in the new towns after the initial excess (Kinlen *et al*., 1990) and of which there have been signs in other studies (Alexander and McKinney, 1990).

### Relevance to the clusters near Sellafield and Dounreay

Now that the theory about paternal preconceptional irradiation (Gardner *et al*., 1990) can be seen to be incorrect (Doll *et al*., 1994), the relevance of the population mixing hypothesis to the excess of leukaemia in Seascale deserves careful examination, particularly given the light it has shed on the cluster near Dounreay (see section e above). Each of the studies reviewed here has concerned areas of isolation (and sometimes also high social class) affected by residential or occupational population mixing. In all these respects Seascale is extreme. Of more than 11 000 rural parishes in England and Wales, few experienced a greater post-war population increase than did Seascale (3-fold between 1948 and 1961), and those that did were not so geographically isolated. It is also an area of exceptionally high social class: in 1961 43% of men were in social class I (Gardner *et al*., 1987), a proportion approached by no other rural growth parish. Furthermore, the adjacent worksite at Sellafield, where most people in Seascale are employed, is highly unusual among rural industrial sites in England. Not only is it probably the largest (with about 9000 'nuclear' employees) but, like the oil industry sites studied in northern Scotland, it has had over the years large numbers of migrant construction workers, a ready source of infection. This aspect of Sellafield over most of its 40 year history makes it unique among rural work sites in Britain (Kinlen *et al*., 1993) and, coupled with the regular supply of new susceptible individuals among the incomers to Seascale, may also explain the protracted nature of the leukaemia excess there. The recent discovery of another significant cluster of childhood leukaemia in nearby Egremont North (Craft *et al*., 1993) is noteworthy since it serves as the local centre for the migrant contractors' workforce.

Strongly suggestive of an infective process is the marked difference in age among the young people with leukaemia and non-Hodgkin's lymphoma from Seascale and the Thurso areas between those born locally and those who moved into those areas later. All eight children affected below age 5, but only three of the 13 older individuals, were born locally (Kinlen, 1993, and unpublished; Kinlen *et al*., 1993). This relative sparing of older individuals who were born locally is consistent with their having been immunised by their earlier exposures.

### Other supporting evidence

An infective origin is supported by the recent demonstration of a modest degree of space–time clustering of childhood leukaemia in a large data set covering Britain in the period 1963–83 (Gilman and Knox, 1991). Several other observations are also consistent with such an origin. Thus, in those parts of rural Scotland that were unaffected by the North Sea oil industry, the disease peaked at age 3 instead of at age 2 as in urban areas (Kinlen *et al*., 1993), a pattern similar to that observed in several childhood infections (Anderson and May, 1982). The significant excess of acute lymphoblastic leukaemia at ages 1–7 in isolated wards of England and Wales that were of high social class in two separate data sets is also suggestive of an infective basis (Alexander *et al*., 1990). The examination of sequential leukaemia incidence and details of such external changes as influxes or commuting was outside the scope of this study even though such influences are not implausible.

The recent report of a significantly lower incidence of leukaemia in Greek children who had attended a creche in infancy raises the possibility that immunising doses of some relevant infection were involved in such early exposures (Petridou *et al*., 1993). If so, a similar explanation may apply to the lower risk among later born than in first-born children, noted in several case–control studies (Stark and Mantel, 1969; Shaw *et al*., 1984). The population mixing hypothesis may also provide some explanation for the most well-known cluster of childhood leukaemia in Niles, IL, USA. The eight cases, seven of them among the pupils of a crowded parish school or in their siblings, occurred during a population influx when the town increased about 5-fold, much of it in that particular parish (Heath and Hasterlick, 1963).

### Implications for future work

It would not be surprising if, in epidemic situations like those summarised above, children with a high level of (direct *and or* indirect) personal contacts were more at risk than those with a low level. Whether such differences would be detectable by case–control studies *outside* such extreme situations (when infection is presumably sporadic) is more uncertain. It will be important to establish this one way or the other for each of the principal cell types. Measuring an individual's 'level' of contacts even crudely is no easy matter, but certain indirect measures might usefully be investigated. Thus, adoption, changes of address and certain occupations of household members must all tend to increase the totality of a child's contacts.

Microbiological assays have great relevance in future case–control studies, for without candidate agents such studies in the usual (non-epidemic) situations may be relatively unhelpful. The model of feline leukaemia shows that multi-cat households offer the only epidemiological signs of transmission unless virus studies are used. Recent work suggests that mathematical models may provide a promising way for understanding patterns of childhood leukaemia at the population level (Langford, 1992).

### Conclusion

The population mixing hypothesis does not imply answers to many specific questions about the infection in question – no more than did, say, the early evidence for a sexually transmitted infection underlying cervix cancer indicate the relative importance of age at first intercourse and the number of partners: these were subjects for special study later. The consistency and magnitude of the excesses found in the studies reviewed here (see Table I) effectively rule out the operation of chance and nor can they be explained by bias or by any known indirect relationship. An infective basis in childhood leukaemia is the only category of causation of a human malignancy that is suggested by these data.

### References

ALEXANDER FE, RICKETTS TJ, MCKINNEY PA AND CARTWRIGHT RA. (1990). Community lifestyle characteristics and risk of acute lymphoblastic leukaemia in children. *Lancet*, **336**, 1461-1465.

ALEXANDER FE AND MCKINNEY PA. (1990). Infectious aetiology of childhood leukaemia. *Lancet*, **336**, 944-945.

ANDERSON RM AND MAY RM. (1982). Directly transmitted infectious diseases; control by vaccination. *Science*, **215**, 1053-1060.

BARTLETT MS. (1960). *Stochastic Population Models in Ecology and Epidemiology*. Methuen: London.

CRAFT AW, PARKER L, OPENSHAW S, CHARLTON M, NEWELL J, BIRCH JM AND BLAIR V. (1993). Cancer in young people in the north of England, 1968-85; analysis by census wards. *Epidemiol. Commun. Hlth.*, **47**, 109-115.

DOLL R, EVANS HJ AND DARBY S. (1994). Paternal exposure not to blame. *Nature*, **367**, 678-680.

GARDNER MJ, HALL AJ, DOWNES S AND TERRELL JD. (1987). Follow up study of children born to mothers resident in Seascale, West Cumbria (birth cohort). *Br. Med. J.*, **295**, 822-827.

GARDNER MJ, SNEE MP, HALL AJ, POWELL CA, DOWNES S AND TERRELL JD. (1990). Results of case-control study of leukaemia and lymphoma among young people near Sellafield nuclear plant in West Cumbria. *Br. Med. J.*, **300**, 423-429.

GILMAN EA AND KNOX EG. (1991). Temporal-spatial distribution of childhood leukaemias and non-Hodgkin lymphomas in Great Britain. In *The Geographical Epidemiology of Childhood Leukaemia and Non-Hodgkin's Lymphoma in Great Britain 1966-83*, Draper G. (ed.) pp. 77-99. OPCS: London.

GREAVES MF. (1988). Speculations on the cause of childhood acute lymphoblastic leukaemia. *Leukaemia*, **2**, 120-125.

HEATH CW AND HASTERLICK RJ. (1963). Leukemia among children in a suburban community. *Am. J. Med.*, **34**, 796-812.

KELLETT CE. (1937). Acute leukaemia in one of identical twins. *Arch. Dis. Child.*, **12**, 239-252.

KINLEN LJ. (1988). Evidence for an infective cause of childhood leukaemia comparison of a Scottish new town with nuclear reprocessing sites in Britain. *Lancet*, **ii**, 1323-1327.

KINLEN LJ, CLARKE K AND HUDSON C. (1990). Evidence from population mixing in British New Towns 1946-85 of an infective basis of childhood leukaemia. *Lancet*, **336**, 577-582.

KINLEN LJ AND HUDSON C. (1991). Childhood leukaemia and poliomyelitis in relation to military encampments in England and Wales in the period of national military service, 1950-63. *Br. Med. J.*, **303**, 1357-1362.

KINLEN LJ, HUDSON CM AND STILLER CA. (1991). Contracts between adults as evidence for an infective origin of childhood leukaemia: an explanation for the excess near nuclear establishments in West Berkshire? *Br. J. Cancer*, **64**, 549-554.

KINLEN LJ. (1992). Childhood leukaemia on Greek Islands. *Lancet*, **339**, 252-253.

KINLEN LJ. (1993). Can paternal preconceptional radiation account for the increase of leukaemia and non-Hodgkin's lymphoma in Seascale? *Br. Med. J.*, **306**, 1718-1721.

KINLEN LJ. (1994). Leukaemia. In *Trends in Cancer Incidence and Mortality*, Doll R, Fraumini JF and Muir CS. (eds) pp. 475-491. Cold Spring Harbor Laboratory Press: Cold Spring Harbor, NY.

KINLEN LJ, O'BRIEN F, CLARKE K, BALKWILL A AND MATTHEWS F. (1993). Rural population mixing and childhood leukaemia: effects of the North Sea oil industry in Scotland, including the area near Dounreay nuclear site. *Br. Med. J.*, **306**, 743-748.

KINLEN LJ AND JOHN SM. (1994). Wartime evacuation of children and mortality from childhood leukaemia in England and Wales in 1945-49. *Br. Med. J.*, **309**, 1197-1201.

KINLEN LJ AND PETRIDOU E. (1994). Childhood leukaemia and Rural-Urban migration in Greece and other countries (submitted).

LANGFORD I. (1991). Chilhood leukaemia mortality and population change in England and Wales 1969-73. *Soc. Sci. Med.*, **33**, 435-440.

LANGFORD I. (1992). Childhood leukaemia and infections. PhD Thesis. University of East Anglia.

LAPLANCHE A AND DE VATHAIRE F. (1994*a*). Leukaemia mortality in French communes (administrative units) with a large and rapid population. *Br. J. Cancer*, **69**, 110-113.

LAPLANCHE A AND DE VATHAIRE F. (1994*b*). Leukaemia mortality in Growth French communes. *Br. J. Cancer*, **70**, 181.

ONIONS DE AND JARRETT O. (1987). Viral oncogenesis lessons from naturally occurring animal viruses. *Cancer Surv.*, **6**, 161-180.

PETRIDOU E, CHUNG CHENG HSIEH, KOTSIFAKIS G, SHAKIDIS Y AND TRICHOPOULOS D. (1991). Absence of leukaemia clustering on Greek Islands. *Lancet*, **338**, 1204-1205.

PETRIDOU E, KASSIMOS D, KALMANTI M, KOSMIDIS H, HAIDAS S, FLYTZANI V, TONG D AND TRICHOPOULOS D. (1993). Age of exposure to infections and risk of childhood leukaemia. *Br. Med. J.*, **307**, 774.

SHAW G, LAVEY R, JACKSON R AND AUSTIN D. (1984). Association of childhood leukaemia with maternal age, birth order and paternal occupation. *Am. J. Epidemiol.*, **119**, 788-795.

SMITH PG. (1982). Spatial and temporal clustering. In *Cancer Epidemiology and Prevention*. Schottenfield D and Fraumeni JR. (eds) pp. 391-407. W.B. Saunders: Philadelphia.

STARK CR AND MANTEL N. (1969). Maternal-age and birth-order effects in childhood leukaemia: age of child and type of leukaemia. *J. Natl Cancer Inst.*, **42**, 857-866.

STOCKS P. (1942). Measles and whooping-incidence before and during the dispersal of 1939-41. *J. R. Stat. Soc.*, **CV**: 259-291.

TOPLEY WWC. (1942). The biology of epidemics. *Proc. R. Soc. Lond.*, **130**, 337-359.

WOLFF SP. (1991). Leukaemia and wartime evacuation. *Nature*, **349**, 23.